# Application of metagenomic next-generation sequencing and targeted metagenomic next-generation sequencing in diagnosing pulmonary infections in immunocompetent and immunocompromised patients

**DOI:** 10.3389/fcimb.2024.1439472

**Published:** 2024-08-06

**Authors:** Yong Liu, Wencai Wu, Yunping Xiao, Hongyan Zou, Sijia Hao, Yanfang Jiang

**Affiliations:** ^1^ Genetic Diagnosis Center, The First Hospital of Jilin University, Changchun, China; ^2^ Key Laboratory of Biomedical Information Engineering of Ministry of Education, Biomedical Informatics & Genomics Center, School of Life Science and Technology, Xi’an Jiaotong University, Xi’an, Shaanxi, China

**Keywords:** pulmonary infection, bronchoalveolar lavage fluid, TNGS, MNGs, immunocompetent and immunocompromised

## Abstract

**Background:**

Metagenomic next-generation sequencing (mNGS) technology has been widely used to diagnose various infections. Based on the most common pathogen profiles, targeted mNGS (tNGS) using multiplex PCR has been developed to detect pathogens with predesigned primers in the panel, significantly improving sensitivity and reducing economic burden on patients. However, there are few studies on summarizing pathogen profiles of pulmonary infections in immunocompetent and immunocompromised patients in Jilin Province of China on large scale.

**Methods:**

From January 2021 to December 2023, bronchoalveolar lavage fluid (BALF) or sputum samples from 546 immunocompetent and immunocompromised patients with suspected community-acquired pneumonia were collected. Pathogen profiles in those patients on whom mNGS was performed were summarized. Additionally, we also evaluated the performance of tNGS in diagnosing pulmonary infections.

**Results:**

Combined with results of mNGS and culture, we found that the most common bacterial pathogens were *Pseudomonas aeruginosa*, *Klebsiella pneumoniae*, and *Acinetobacter baumannii* in both immunocompromised and immunocompetent patients with high detection rates of *Staphylococcus aureus* and *Enterococcus faecium*, respectively. For fungal pathogens, *Pneumocystis jirovecii* was commonly detected in patients, while fungal infections in immunocompetent patients were mainly caused by *Candida albicans*. Most of viral infections in patients were caused by Human betaherpesvirus 5 and Human gammaherpesvirus 4. It is worth noting that, compared with immunocompetent patients (34.9%, 76/218), more mixed infections were found in immunocompromised patients (37.8%, 14/37). Additionally, taking final comprehensive clinical diagnoses as reference standard, total coincidence rate of BALF tNGS (81.4%, 48/59) was much higher than that of BALF mNGS (40.0%, 112/280).

**Conclusions:**

Our findings supplemented and classified the pathogen profiles of pulmonary infections in immunocompetent and immunocompromised patients in Jilin Province of China. Most importantly, our findings can accelerate the development and design of tNGS specifically used for regional pulmonary infections.

## Introduction

Although some progress has been made in the treatment of pulmonary infections, pulmonary infection is still an important cause of mortality worldwide (Ravimohan et al., 2018; D'Anna et al., 2021; Schneider et al., 2021). Therefore, early identification of pathogens is crucial for rapid clinical diagnosis and initial treatment. Although there are various detection methods, it is still a challenge to quickly and accurately diagnose pulmonary infections (Luyt et al., 2020; Turcios, 2020). Conventional methods, including microbial culture (Handel et al., 2021), microscopic smear (Yue et al., 2021), polymerase chain reaction (PCR) (Ramachandran and Wilson, 2020), nucleic acid hybridization, histopathology (Greninger and Naccache, 2019), and serological antibody detection (Simner et al., 2018), can only identify about 40% of pathogens, with long detection time and low positive detection rate, which cannot meet the current clinical needs (Qu et al., 2022; Shi et al., 2022). Besides, empirical therapy is often ineffective in treating immunocompromised patients with atypical pneumonia (Azoulay et al., 2019). Therefore, rapid and accurate identification of pathogens is very important for the clinical intervention of these patients.

At present, the metagenomic next-generation sequencing (mNGS) is a new and rapidly developing pathogen diagnosis technology (Han et al., 2019). mNGS has the advantages of short detection time and wide detection range (Gökdemir et al., 2022), which can accurately identify bacteria, fungi, viruses, parasites, and other pathogens (Indelli et al., 2021). Clinicians has extensively used mNGS to diagnose various infections, such as bloodstream infection, abdominal cavity infection, central nervous system infection, etc (Greninger and Naccache, 2019; Thakur, 2020; Li et al., 2022). mNGS has become a promising detection method for infectious diseases (Zhou et al., 2022).

Recently, based on the most common pathogen profiles, targeted mNGS (tNGS) using multiplex PCR (Xie et al., 2022), reducing economic burden on patients, has been developed to detect pathogens with predesigned primers in the panel (Li et al., 2021; Huang et al., 2023). However, the occurrence of infections in immunocompromised patients caused by rare (Zhan et al., 2021), regional (Ramirez et al., 2020), and emerging (El Zein et al., 2020; Fishman, 2023) pathogens limited the application of this tNGS in those patients. However, there are few studies on summarizing pathogen profiles of pulmonary infections in immunocompetent and immunocompromised patients in Jilin Province of China on large scale.

Given the advantage of tNGS and comprehensiveness of mNGS, it is necessary to summarize pathogen profiles of pulmonary infections in Jilin Province of China using mNGS to provide reference for designing and developing regional tNGS. Therefore, our study retrospectively enrolled immunocompetent and immunocompromised patients with suspected community-acquired pneumonia, evaluated the value of mNGS in the diagnosis of pulmonary infections by comparing the diagnostic performance of mNGS and conventional culture using sputum and bronchoalveolar lavage fluid (BALF) samples, and summarized pathogen profiles in both immunocompetent and immunocompromised patients. Additionally, we also summarized the coincidence rate of tNGS results against final comprehensive clinical diagnoses.

## Methods

### Ethics statement

This study was reviewed and approved by the Ethical Review Committee of the First hospital of Jilin University (approval no. 2024–612). All procedures followed were in strict compliance with the Ethical Review of Biomedical Research Involving Human Subjects (2016), the Declaration of Helsinki, and the International Ethical Guidelines for Biomedical Research Involving Human Subjects. The study was exempted from requiring informed consent by the Ethical Review Committee as it was a retrospective study and patient data were anonymized.

### Patients and sample collection

**Figure 1:**
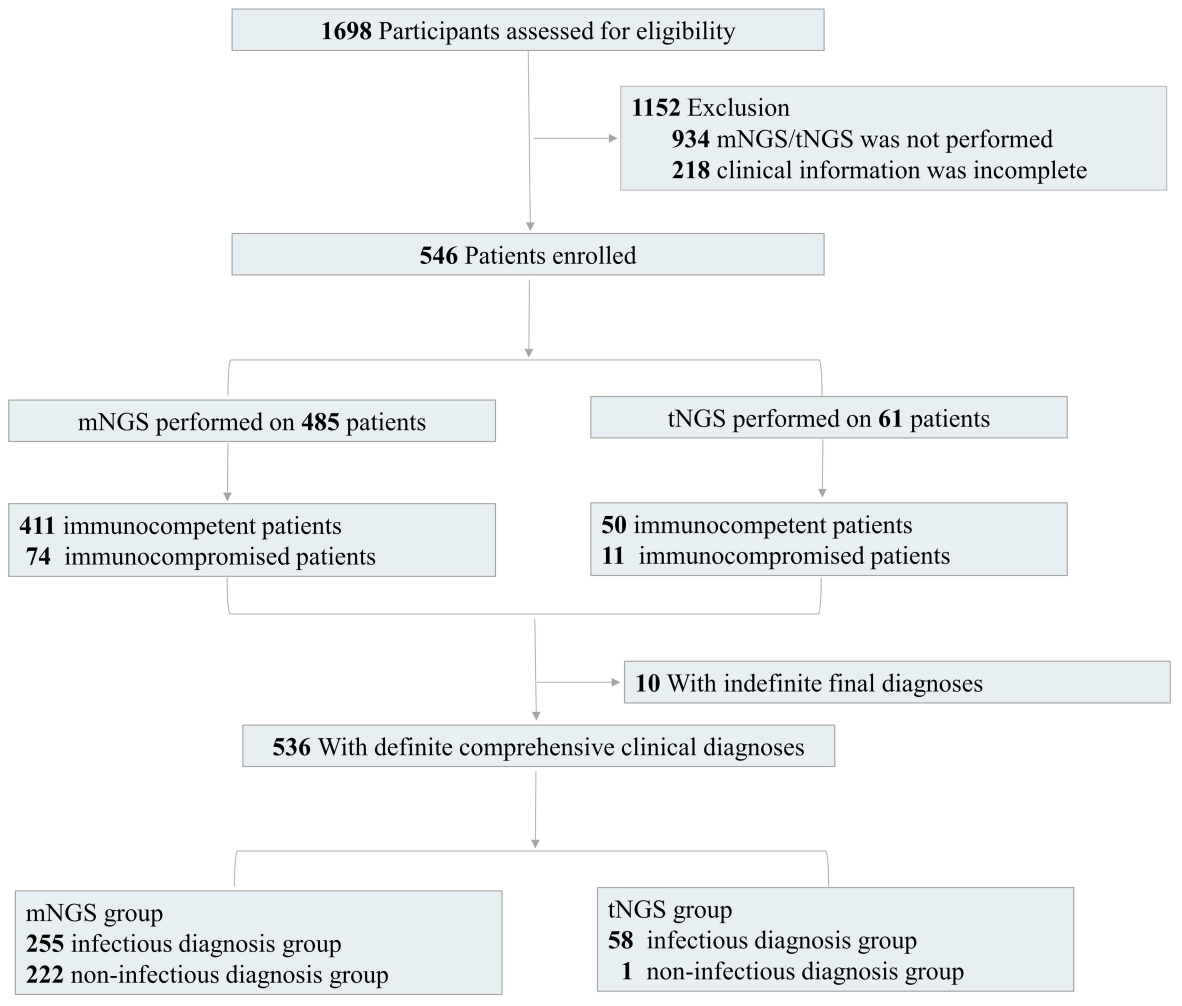
Flow diagram. We retrospectively assessed the eligibility of patients. According to the inclusion and exclusion criteria, we finally enrolled 546 patients with detection results of mNGS/tNGS and culture and complete clinical information, including 485 patients with mNGS results and 61 patients with tNGS results. We also divided the enrolled patients into immunocompetent and immunocompromised groups. There were 10 patients with indefinite final diagnoses.

A total of 546 patients admitted to The First Hospital of Jilin University from January 2021 to December 2023 and diagnosed as suspected community-acquired pneumonia were retrospectively enrolled (**Figure 1**). Specimens were subjected to conventional culture and mNGS/tNGS testing in parallel. All patients with suspected pulmonary infection had abnormal chest imaging results. The inclusion criteria were as follows: (1) Patients with symptoms such as fever, cough, sputum, dyspnea, and imaging abnormalities, such as lung shadows, space-occupying lesions, and other signs of pulmonary infection, (2) Patients on whom mNGS/tNGS was performed, and (3) Patients with complete clinical data. The exclusion criteria were as follows: (1) Patients on whom mNGS/tNGS was not performed and (2) Patients with incomplete clinical and laboratory data.

### mNGS pipeline

BALF and sputum samples were collected based on the standard clinical procedure. After collection, BALF or sputum samples were immediately inactivated at 65°C for 30 minutes. A 1.5 mL microcentrifuge tube with 0.5 mL sample and 1 g 0.5 mm glass beads was attached to a horizontal platform on a vortex mixer and agitated vigorously at 2800–3200 rpm for 30 min. Subsequently, 0.3 mL supernatant sample was separated into a new 1.5 mL microcentrifuge tube and DNA was extracted using the TIANamp Micro DNA Kit (DP316, TIANGEN BIOTECH) according to the manufacturer’s recommendation. Qubit 4.0 (Thermo Fisher Scientific, MA, USA) was used to measure extracted DNA concentrations. QIAseq Ultralow Input Library Kit (QIAGEN, Hilden, Germany) was used to construct metagenomics libraries (Ji et al., 2020). Inspected and qualified library was sequenced on Nextseq 550 platform (Illumina, San Diego, USA).

### Commercial tNGS pipeline

Based on multiplex PCR and mNGS, in-house tNGS panel was designed to detect 273 pathogens, including 113 bacteria, 47 fungi, 101 viruses, and 12 parasites, causing infections in different systems according to the public pathogen databases and the published studies. After nucleic acid extraction, multiplex PCR with the designed primers was used to construct libraries. Library concentrations were quantified using Qubit 4.0 and Illumina NextSeq platform was used for high-throughput sequencing.

### Analysis of sequencing data

Raw sequencing data were exposed to quality control, including the removal of low-quality reads (Q score cutoff, 20). High-quality sequencing data were generated using in-house software, followed by computational subtraction of human host sequences mapped to the human reference genome (hg38) using Burrows-Wheeler Alignment (Li and Durbin, 2009). The remaining data were blasted against in-house classification reference databases, which were constructed according to the published microbial genome databases, including reference sequence database at National Center for Biotechnology Information. The constructed databases contain 25,863 pathogens, including 12,142 bacteria, 2,680 fungi, 10,061 viruses (including DNA and RNA viruses), 654 parasites, 206 mycobacteria, and 120 mycoplasma/chlamydia.

### Interpretation of mNGS/tNGS results and diagnostic assessment

As control, negative and positive controls were also set with the same procedure and bioinformatics analysis. Strictly map reads number (SMRN) and genomic coverage were analyzed. SMRN represents the number of sequences that are strictly aligned with the microorganism at species level.

During the interpretation process, positive mNGS/tNGS results were defined as follows:

1) Bacteria, fungi, parasites, mycoplasmas, and chlamydiae: When the microorganism was not detected in the negative control (‘No template’ control, NTC) and genome coverage of detected sequences belonged to this microorganism ranked top10 among the microbes in the same genus or when its ratio of SMRN_sample_ to SMRN_NTC_ was > 10 if the SMRN_NTC_≠0. Besides, the SMRN_sample_ of bacteria, fungi, mycoplasmas, and chlamydiae should be ≥ 3, while the SMRN_sample_ of parasites should be ≥ 100. SMRN_sample_ of *Mycobacterium tuberculosis* should be ≥ 1.

2) Viruses: When the virus was not detected in NTC or when SMRN_sample_/SMRN_NTC_ was > 5 if the SMRN_NTC_≠0. Besides, SMRN_sample_ of viruses should be ≥ 1.

Subsequently, positive mNGS/tNGS results were further defined according to whether the detected microbes by mNGS/tNGS were the most commonly reported pathogens or the infections caused by the microbes were in accordance with clinical features of patients.

For diagnostic assessment, 2–3 clinical adjudicators independently made the final comprehensive clinical diagnoses and defined the causative pathogens, according to clinical characteristics, laboratory examinations, response of the patients to treatment, mNGS/tNGS results, and clinical experiences. Based on final comprehensive clinical diagnoses, we divided the enrolled patients into infectious diagnosis group, non-infectious diagnosis group, and indefinite clinical diagnosis group, which were used as reference standard to evaluate the performance of mNGS/tNGS.

The definition of infectious diagnoses was based on 1) At least one of culture result, clinical characteristics, or clinical experiences suggested pulmonary infections, or 2) For the patients without any laboratory examinations or with negative laboratory examinations, pneumonia was relieved after treatment according to the mNGS/tNGS results. The definition of non-infectious diagnoses was based on 1) No pathogens were detected by both culture and mNGS/tNGS, and 2) The inflammation was relieved after the use of glucocorticoid or immunosuppressant. Indefinite clinical diagnosis group included patients whose clinical characteristics or laboratory examinations were not adequate for diagnoses and patients lost during follow-up duration.

Subsequently, causative pathogens can be defined. The non-infectious diagnosis group was used to evaluate the specificity of mNGS, tNGS and culture. In addition, we can accurately divide the detection results into true-positive, false-negative, false-positive, and true-negative results. Guided by mNGS/tNGS results, we also adjusted the therapeutic regimens, which was approved by the Ethical Review Committee.

### Statistical analysis

Continuous variables were expressed as mean ± standard deviation (SD) and categorical variables were presented as numbers (percentage). We tested for differences in continuous variables using t test and categorical variables with chi-square test as appropriate. The 2 × 2 contingency tables were established to determine sensitivity, specificity, and total coincidence rate (TCR), and the results are presented with 95% confidence intervals (CIs). Data analyses were performed using SPSS 22.0 software and a two-tailed value of *P* of < 0.05 was considered to represent a significant difference.

### Data availability

Sequencing data were deposited to the National Genomics Data Center under accession numbers CRA016476 and CRA017510. The authors declare that the main data supporting the findings are available within this article. The other data generated and analyzed for this study are available from the corresponding author upon reasonable request.

## Results

### Baseline characteristics of enrolled patients

A total of 546 patients [325 males (59.5%) and 221 females (40.5%)] with suspected community-acquired pulmonary infection were included in this study (**Table 1**). Most of the patients were over 40 years old (72.0%), while the patients aged from 40 to 70 years old accounted for 46.7% of the enrolled patients. Among them, 134 (24.5%) patients had underlying diseases, including diabetes (*n* =42, 7.7%), lung cancer (*n* =22, 4.0%), previous history of tuberculosis (*n* =18, 3.3%), bronchiectasis (*n* =16, 2.9%), chronic obstructive pulmonary disease (*n* =14, 2.6%), extrapulmonary malignancies (*n* =11, 2.0%), bronchial asthma (*n* =9, 1.6%), and connective tissue disease (*n* =2, 0.4%).

**Table 1:** Baseline characteristics of 546 patients.

	Number of cases	Percentage (%)
Sex		
Male	325	59.5
Female	221	40.5
Age (years)		
≤ 40	153	28.0
>40, ≤70	255	46.7
> 70	138	25.3
Underlying illness		
Bronchiectasis	16	2.9
Chronic obstructive pulmonary disease	14	2.6
Previous history of tuberculosis	18	3.3
Bronchial asthma	9	1.6
Lung cancer	22	4.0
Diabetes	42	7.7
Connective tissue disease	2	0.4
Extrapulmonary malignancies	11	2.0
Sample type		
BALF	345 (284 for mNGS, 61 for tNGS)	63.2
Sputum	201 (all for mNGS)	36.8

BALF, bronchoalveolar lavage fluid.

Additionally, 284 BALF and 201 sputum samples for mNGS test were respectively collected from 485 patients, including 411 immunocompetent patients and 74 immunocompromised patients. The tNGS test was performed on BALF samples from the other 61 patients, including 50 immunocompetent patients and 11 immunocompromised patients (**Figure 1**). The immunodeficiency was mainly caused by the above underlying diseases, including diabetes, lung cancer, extrapulmonary malignancies, connective tissue disease, or the other diseases with long-term immunosuppressive treatment.

### Comparison between mNGS and culture

**Figure 2:**
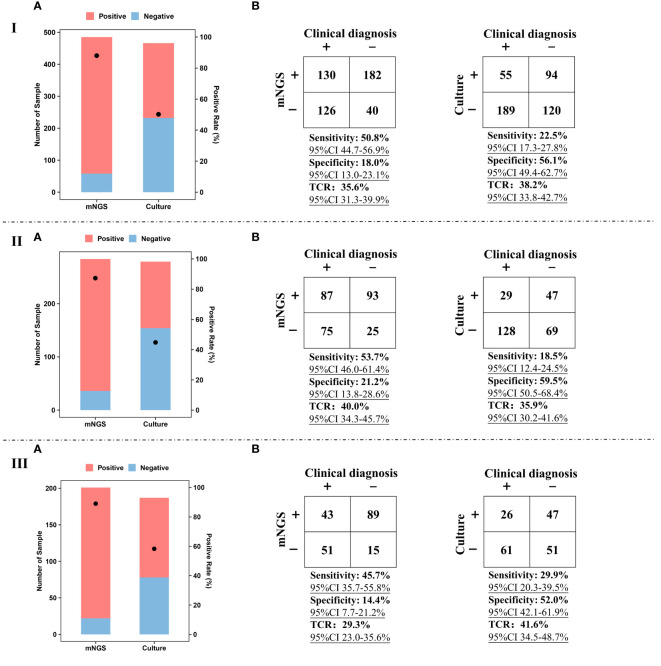
Performance of mNGS and culture. I, Positive rate **(A)**, sensitivity, specificity, and TCR **(B)** of mNGS and culture using BALF and sputum samples. II, Positive rate **(A)**, sensitivity, specificity, and TCR **(B)** of mNGS and culture using BALF samples. III, Positive rate **(A)**, sensitivity, specificity, and TCR **(B)** of mNGS and culture using sputum samples. The ‘TCR’ is total coincidence rate, and ‘CI’ represents confidence interval.

mNGS and culture were simultaneously performed on BALF and sputum samples and the diagnostic value of mNGS and culture using different kinds of samples was summarized (**Figure 2**). For BALF and sputum samples, the positive rate of mNGS can reach up to 88.0%, which was much higher than that of culture (50.2%) (**Figure 2IA**). For BALF samples, the positive rate of mNGS (87.3%) was much higher than that of culture (44.8%) (**Figure 2IIA**). For sputum samples, a similar trend (89.1% vs 58.3%) was found (**Figure 2IIIA**).

For BALF and sputum samples, sensitivity [50.8% (95% CI 44.7–56.9%)] of mNGS was much higher than that of culture [22.5% (95% CI 17.3–27.8%)], taking final comprehensive clinical diagnoses as reference standard (**Figure 2IB**). The specificity of culture can reach up to 56.1%, while the specificity of mNGS was only 18.0% (95% CI 13.0–23.1%). Besides, there was no significant difference in TCR between mNGS [35.6% (95% CI 31.3–39.9%)] and culture [38.2% (95% CI 33.8–42.7%)]. Similar trends were found in the comparison between BALF mNGS and BALF culture (**Figure 2IIB**) or sputum mNGS and sputum culture (**Figure 2IIIB**). In addition, the sensitivity [53.7% (95% CI 46.0–61.4%)] and TCR [40.0% (95% CI 34.3–45.7%)] of BALF mNGS were higher than those of sputum mNGS [45.7% (95% CI 35.7–55.8%) and 29.3% (95% CI 23.0–35.6%)]. The above results indicate that compared with sputum sample, BALF sample was preferred sample for mNGS in detecting pathogens of pulmonary infections.

### mNGS in immunocompetent and immunocompromised patients with pulmonary infections

**Figure 3:**
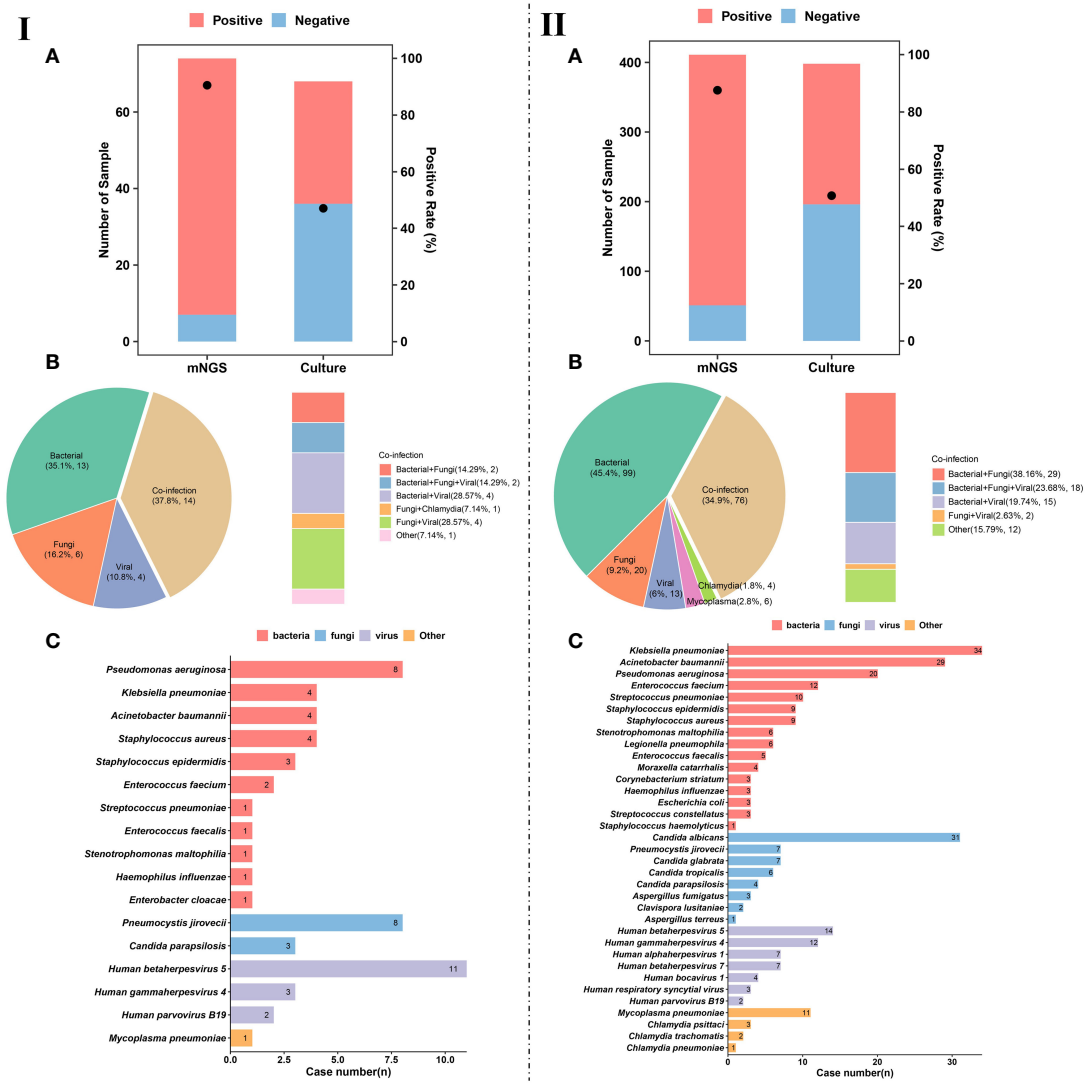
Pathogen detection and characteristics of pulmonary infection. **(IA)**, Number of patients and positive rate of mNGS and culture in immunocompromised patients. **(IIA)**, Number of patients and positive rate of mNGS and culture in immunocompetent patients. **(IB)**, Different kinds of infections (*n* = 37) in immunocompromised patients. **(IIB)**, Different kinds of infections (*n* = 218) in immunocompetent patients. **(IC)**, Pathogen profiles in immunocompromised patients. **(IIC)**, Pathogen profiles in immunocompetent patients.

We divided the enrolled patients on whom mNGS was performed into immunocompetent (*n* = 411) and immunocompromised (*n* = 74) groups, and the diagnostic positive rates of mNGS and conventional culture in the two groups were calculated (**Figure 3**). It was found that the performance of mNGS was significantly higher than that of culture in both immunocompetent and immunocompromised groups. However, there was no significant difference in the positive rate of mNGS between immunocompromised (90.5%) (**Figure 3IA**) and immunocompetent (87.6%) (**Figure 3IIA**) groups (*P*>0.05). A similar trend was found in the positive rate of culture (47.1% and 50.8%) (*P*>0.05).

Among the infected patients in immunocompromised group, single infection accounted for 62.1% (23/37). For single infection, the most common pathogens were bacteria (35.1%, 13/37), followed by fungi (16.2%, 6/37) and viruses (10.8%, 4/37) (**Figure 3IB**). In addition to single infection, our results show that the number of patients with mixed infection accounted for 37.8% (14/37) of immunocompromised patients, including bacterial-fungal co-infection (*n* = 2), bacterial-viral co-infection (*n* = 4), fungal-viral co-infection (*n* = 4), and bacterial-fungal-viral co-infection (*n* = 2) (**Figure 3IB**).

The pathogen profiles of immunocompromised patients were summarized (**Figure 3IC**). We found that *Pseudomonas aeruginosa* (*n* = 6), *Klebsiella pneumoniae* (*n* = 4), *Acinetobacter baumannii* (*n* = 4), and *Staphylococcus aureus* (*n* = 4) were the most common bacterial pathogens in immunocompromised patients with pulmonary infection. The most common fungal pathogens were *Pneumocystis jirovecii* (*n* = 8) and *Candida parapsilosis* (*n* = 3). Besides, viral infection was mainly caused by Human betaherpesvirus 5 (CMV) (*n* = 11), Human gammaherpesvirus 4 (EBV) (*n* = 3), and human parvovirus B19 (*n* = 2).

For the infected patients in immunocompetent group, single infection accounted for 65.1% (142/218) (**Figure 3IIB**). For single infection, the most common pathogens were bacteria (45.4%, 99/218), followed by fungi (9.2%, 20/218), and viruses (6%, 13/218) (**Figure 3IIB**). In addition to single infection, our results show that mixed infection occurred in 34.9% (76/218) of immunocompetent patients, such as bacterial-fungal co-infection (*n* = 29), bacterial-fungal-viral co-infection (*n* = 18), bacterial-viral co-infection (*n* = 15), and fungal-viral co-infection (*n* = 12) (**Figure 3IIB**). It is worth noting that more mixed infections are found in immunocompromised patients than in immunocompetent patients.

The pathogen profiles of were also summarized (**Figure 3IIC**). We found that the most common bacterial pathogens were *K. pneumoniae* (*n* = 34), *A. baumannii* (*n* = 29), *P. aeruginosa* (*n* = 20), and *Enterococcus faecium* (*n* = 12). Fungal infection in immunocompetent patients with pulmonary infection was mainly caused by *Candida albicans* (*n* = 31), *P. jirovecii* (*n* = 7), and *Candida glabrata* (*n* = 7). The most common viral pathogens were CMV (*n* = 14), EBV (*n* = 12), Human alphaherpesvirus 1 (*n* = 7). At the same time, we also detected *Mycoplasma pneumoniae*, *Chlamydia psittaci*, *Chlamydia trachomatis*, and Chlamydia pneumoniae from 11, 3, 2, and 1 patients, respectively.

### Adjustment of antibiotics and prognosis

**Figure 4:**
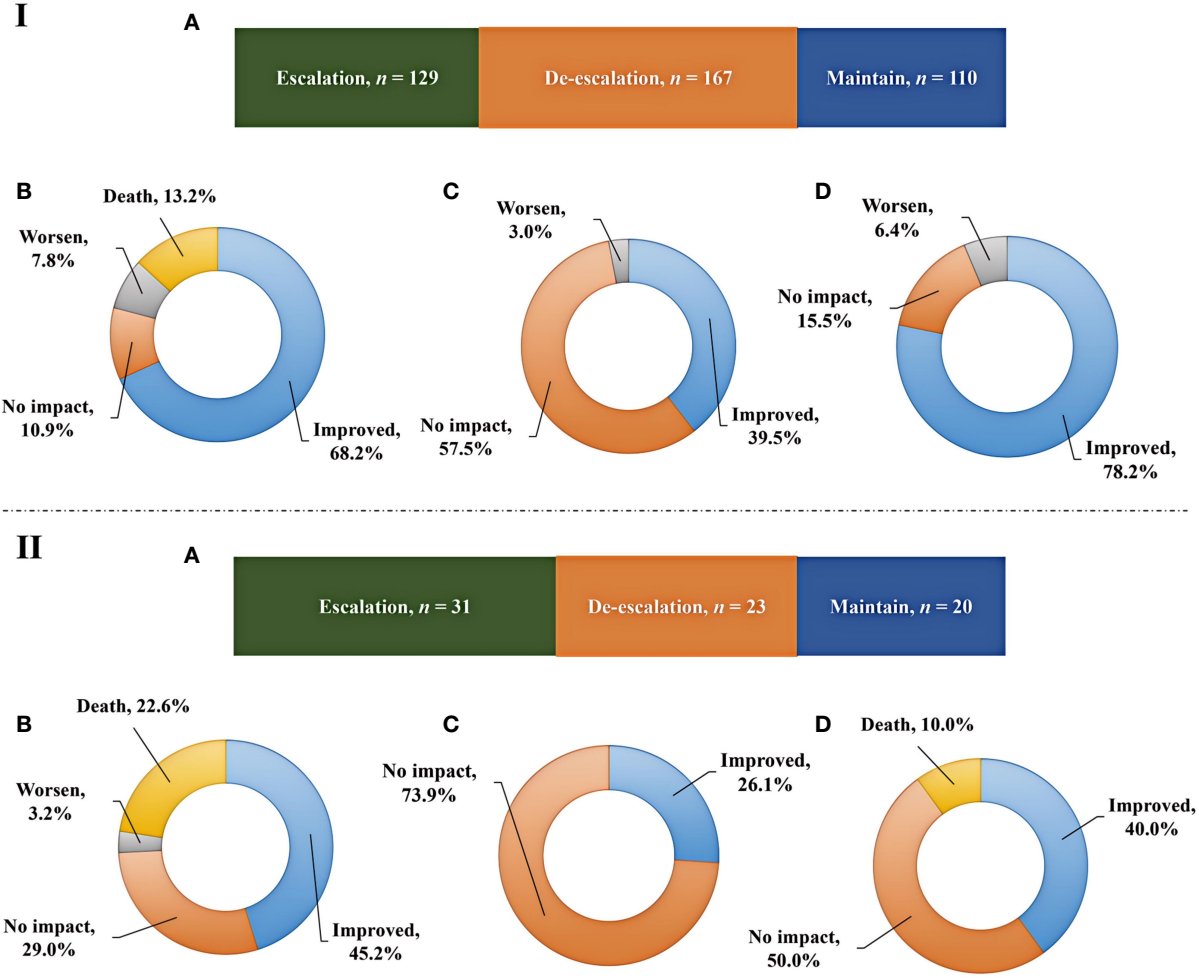
Adjustment of antibiotics and prognosis. **(IA, IIA)**, Impact of mNGS on adjustments of antibiotics in immunocompetent and immunocompromised patients, respectively. **(IB, IC, ID)**, Outcomes among immunocompetent patients receiving escalation, de-escalation, and maintain treatments, respectively. **(IIB, IIC, IID)**, Outcomes among immunocompromised patients receiving escalation, de-escalation, and maintain treatments, respectively.

mNGS plays an important role in providing reference for clinical therapy. The effects of mNGS on adjustment of antibiotics in both immunocompromised and immunocompetent patients were summarized (**Figure 4**). mNGS exhibited positive impact on most of patients, including 41.1% of immunocompetent patients (*n* = 167) and 31.1% of immunocompromised patients (*n* = 23) receiving de-escalation treatment and 31.8% of immunocompetent patients (*n* = 129) and 41.9% of immunocompromised patients (*n* = 31) receiving escalation treatment (**Figures 4IA, IIA**). In addition, the empirical treatment on the 27.1% of immunocompetent patients (*n* = 110) and 27% of immunocompromised patients (*n* = 20) was not changed.

Although prognosis is influenced by a variety of complex factors, we have summarized the outcomes among patients receiving escalation, de-escalation, and maintain treatments (**Figure 4**). Escalation treatment did not result in improved prognosis in all of patients. Only 68.2% of immunocompetent patients and 45.2% of immunocompromised patients receiving escalation treatment had improved outcome, while deaths accounted for 13.2% and 22.6% of cases, respectively (**Figures 4IB, IIB**). In addition, de-escalation treatment had no impact on most of patients, including 57.5% of immunocompetent patients and 73.9% of immunocompromised patients, and no patients died (**Figures 4IC, IIC**). The highest proportion of patients showing improved prognosis was observed in immunocompetent individuals receiving maintain treatment, while 10.0% of immunocompromised patients died (**Figures 4ID, IID**).

### Application of commercial tNGS in clinics

**Figure 5:**
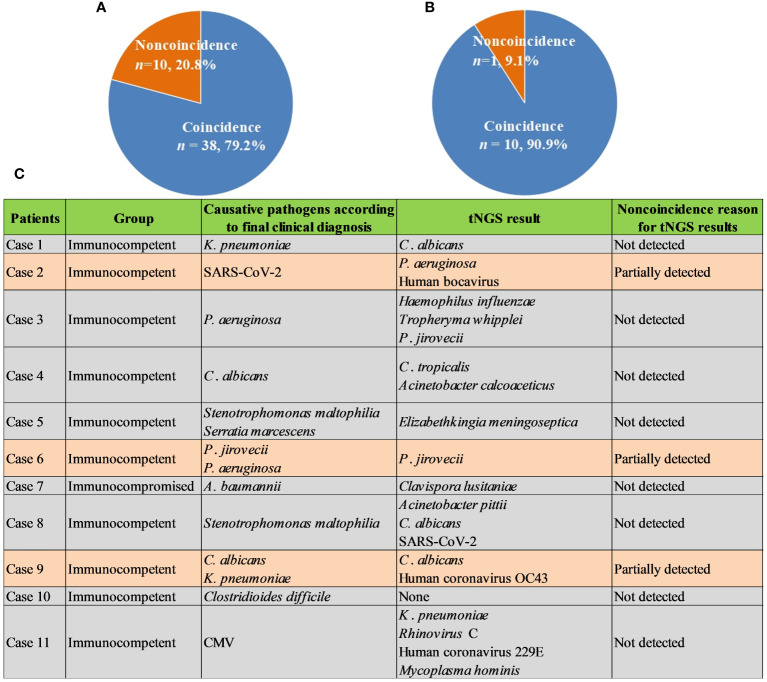
Performance of tNGS. **(A)**, Coincidence rate of tNGS against final comprehensive clinical diagnoses in immunocompetent patients. **(B)**, Coincidence rate of tNGS against final comprehensive clinical diagnoses in immunocompromised patients. **(C)**, Noncoincidence reason for tNGS results.

Given high cost of mNGS and its positive impact on patients, we further explored the role of commercial tNGS in diagnosing pulmonary infections in clinics. It was found that the coincidence rates of tNGS against final comprehensive clinical diagnoses in immunocompetent and immunocompromised patients can reach up to 79.2% (38/48) (**Figure 5A**) and 90.9% (10/11) (**Figure 5B**), respectively, which were much higher than that of BALF mNGS (**Figure 2IIB**). Further analysis of noncoincidence between tNGS and final comprehensive clinical diagnoses (**Figure 5C**) showed that some causative pathogens were partially detected or not detected by tNGS. Although commercial tNGS can provided much more accurate clinical reference for clinics, further improvement, such as optimizing the primer pool or designing a regional-specific tNGS, can be further implemented to enhance the suitability of tNGS for regional pulmonary infections, based on our findings of this study.

## Discussion

This is a retrospective study on evaluating the diagnostic performance of mNGS and tNGS in detecting pulmonary infections in immunocompetent and immunocompromised patients. The results showed that the overall positive rate of mNGS in detecting pathogens of pulmonary infections was significantly higher than that of culture (*P*<0.05) in both immunocompetent and immunocompromised patients. This result is consistent with previous study that the proportion of clinically relevant pathogens found by mNGS was significantly higher than that found by conventional methods (Joensen et al., 2017). Compared with the current conventional microbial methods, mNGS has more advantages in microbial detection (Joensen et al., 2017). The above findings, together with our results, confirm that mNGS can improve the detection rate of pathogens, which is critical to the accurate and rapid diagnosis of pulmonary infection and to guiding the treatment and prevention of patients.

In addition, this study compared the positive detection rates of mNGS and culture not only between BALF and sputum samples but also between immunocompetent and immunocompromised patients. We found that mNGS detection was superior to conventional culture in detecting bacteria, fungi, mycoplasma, and viruses, improving the clinical guidance on the use of antibiotics. This is consistent with the research of Langelier et al. that the detection rate of pathogens in HSCT patients with acute respiratory diseases detected by BALF mNGS was higher than that detected by conventional methods (Langelier et al., 2018). In addition, BALF and sputum mNGS had higher sensitivity (53.7% vs. 18.5% and 45.7% vs. 29.9%, *p*<0.001), but lower specificity (21.2% vs. 59.5% and 14.4% vs. 52.0%, *p*<0.001) than culture, which was consistent with the result of Wang et al. that compared with conventional tests, mNGS has a higher sensitivity in detecting pathogens of lung infection (Wang et al., 2019). Huang et al. also proposed that conventional pathogen detection methods have limitation in accurately and comprehensively detecting microorganisms (Huang et al., 2020).

Increasing evidences suggest that mNGS is an important means for clinical diagnosis of infectious diseases (He et al., 2022; Yu et al., 2022; Qian et al., 2023; Zhang et al., 2023). The comparison between the immunocompetent and immunocompromised groups showed that although the positive rates of mNGS detection were higher than those of the conventional detection, there was no significant difference in the positive rates between the two groups. Besides, compared with patients with normal immune function, more mixed infections were found in patients with immunodeficiency. In conclusion, mNGS is superior to conventional pathogen detection in etiological diagnosis of pulmonary infection, especially for the immunocompromised patients.

As far as we know, mNGS is often used in clinical practice for etiological diagnosis of pulmonary infection and reasonable use of antibiotics. Li et al. showed that mNGS has the advantage in diagnosing mixed pulmonary infection of immunocompromised patients, which is crucial for the accurate diagnosis (Li et al., 2020). In our study, mNGS exhibited positive impact on most of patients, including 73.0% of immunocompromised patients and 72.9% of immunocompetent patients. The abuse and unreasonable use of antibiotics will make more opportunistic lung infections and become a thorny clinical problem for immunocompromised patients (Bouglé et al., 2022; Griffith and Daley, 2022; Smith et al., 2022). Overuse of antibiotics in immunocompromised patients will lead to drug resistance, while reasonable use of antibiotics can reduce the waste of medical resources (Ramirez et al., 2020; Martin-Loeches et al., 2022). mNGS can help clinicians evaluate empiric antibacterial treatment more comprehensively and effectively adjust therapeutic regimens of the immunocompromised patients with pulmonary infections.

Theoretically speaking, tNGS integrating the high sensitivity of PCR and the high throughput of mNGS can successfully enrich pathogens with predesigned primers in the panel (Li et al., 2021; Huang et al., 2023). However, some most common pathogens were detected in some cases but not detected or partially detected in other cases using the same commercial tNGS pipeline in our study. Actually, multiplex PCR scaling to large panels for broad range of most common pathogens causes the nonlinear increase of primer dimer species that reduces tNGS mapping rates (Xie et al., 2022) and subsequently decreases its performance. Additionally, due to the epidemiology of pathogens characterized by geographical specificity (Ramirez et al., 2020), rarity (Zhan et al., 2021), and novelty (El Zein et al., 2020; Fishman, 2023), commercial tNGS cannot fully meet the requirements of regional pathogen detection. Accordingly, based on our pathogen profiles summarized on a large scale, designing and developing of regional tNGS can be conducted according to the published studies (Xie et al., 2022; Xia et al., 2023).

### Limitation

At the same time, it is worth mentioning that this study also has some limitations. First of all, only sputum and BALF samples were used to evaluate the performance of mNGS or tNGS, and future study should include other samples, such as blood sample. Comparison of mNGS using various body fluid samples to analyze the lung infection may provide more valuable information. Secondly, since there were few negative cases with non-infectious diseases, there might be slight bias in calculating specificity. Thirdly, mNGS of both DNA and RNA should be simultaneously performed to detect comprehensive causative pathogens.

## Conclusion

Our research unravels that mNGS has the advantages of being faster and more sensitive than conventional pathogen culture method in patients with pulmonary infection. In addition, mNGS can identify more mixed infections in both immunocompetent and immunocompromised patients. Additionally, BALF tNGS exhibited better performance than BALF mNGS. Both mNGS and tNGS can identify the pathogen of pulmonary infection as early as possible, help clinicians adjust the treatment of antibiotics in time, and greatly improve the diagnosis of suspected pulmonary infection. Given high cost of mNGS and high sensitivity of tNGS, future development of pathogen molecular detection may focus on designing and developing of regional tNGS.

## Data availability statement

The datasets presented in this study can be found in online repositories. The names of the repository/repositories and accession number(s) can be found below: https://ngdc.cncb.ac.cn/search/all?&q=CRA016476, https://ngdc.cncb.ac.cn/search/all?q=CRA017510, CRA016476 and CRA017510.

## Ethics statement

The studies involving humans were approved by Ethical Review Committee of the First hospital of Jilin University. The studies were conducted in accordance with the local legislation and institutional requirements. The human samples used in this study were acquired from A total of 546 patients admitted to The First Hospital of Jilin University from January 2021 to December 2023 diagnosed as suspected pulmonary infection were retrospectively enrolled, and we only summarized the detection results of those patients. All of the detection using human samples had been finished and we did not do any experiments using the human samples in this study. Written informed consent for participation was not required from the participants or the participants’ legal guardians/next of kin in accordance with the national legislation and institutional requirements.

## Author contributions

YL: Conceptualization, Formal Analysis, Writing – original draft, Writing – review & editing. WW: Formal Analysis, Visualization, Writing – original draft. YX: Methodology, Writing – original draft. HZ: Methodology, Writing – original draft. SH: Data curation, Methodology, Writing – original draft. YJ: Conceptualization, Formal Analysis, Funding acquisition, Methodology, Project administration, Supervision, Writing – original draft, Writing – review & editing.
