# Supplementary material for: Application of metagenomic next-generation sequencing and targeted metagenomic next-generation sequencing in diagnosing pulmonary infections in immunocompetent and immunocompromised patients
Source: Front Cell Infect Microbiol. 2024 Aug 6;14:1439472. doi: 10.3389/fcimb.2024.1439472 (PMC11333343; doi:10.3389/fcimb.2024.1439472)
Supplement: Supplementary file 1 [file DataSheet_1.pdf]

Application of metagenomic next-generation sequencing and targeted metagenomic next-generation sequencing in diagnosing pulmonary infections in immunocompetent and immunocompromised patients

Yong Liu[a], Wencai Wu[b], Yunping Xiao[a], Hongyan Zou[a], Sijia Hao[a], Yanfang Jiang[#a]

[a]Genetic Diagnosis Center, The First Hospital of Jilin University, Changchun 130021, China.

[b]Key Laboratory of Biomedical Information Engineering of Ministry of Education, Biomedical Informatics & Genomics Center, School of Life Science and Technology, Xi'an Jiaotong University, Xi'an, Shaanxi 710049, China

Correspondence: Yanfang Jiang, Ph.D., The First Hospital of Jilin University, Changchun 130021, China; Tel: 86-13756660113; Fax: 86-0431-81879185; E-mail: yanfangjiang@hotmail.com, yanfangjiang@jlu.edu.cn.

Characteristics of pulmonary infection and mNGS detection

In this study, causative pathogens were defined according to the results of mNGS and culture methods, clinical symptoms, other laboratory results, and clinical experience. Patients with pulmonary infection was divided into single infection (*n* = 166) and mixed infection (*n* = 87) groups, and the former accounted for about two thirds of cases. For single infection, bacteria (44.3%, 112/255) were the most common pathogens, followed by fungi (10.7%, 27/255) and viruses (6.7%, 27/255) (Figure S1a). In addition to single infection, our results show that patients with pulmonary infection can also be simultaneously infected by different kinds of pathogens, including bacterial and fungal co-infection, bacterial and viral co-infection, fungal and viral co-infection, and bacterial, fungal and viral co-infection (Figure S1a).

Besides, mNGS detection results are summarized in Figure S1b. We found that the most common bacterial pathogens detected by mNGS were *Klebsiella pneumoniae* (*n* = 69), *Acinetobacter baumannii* (*n* = 47), *Enterococcus faecium* (*n* = 39), and *Staphylococcus epidermidis* (*n* = 38). The most common fungal pathogens were *Candida albicans* (*n* = 116), *Candida parapsilosis* (*n* = 28), *Pneumocystis jirovecii* (*n* = 24), *Candida tropicalis* (*n* = 24), and *Candida glabrata* (*n* = 23). Human gammaherpesvirus 4 (EBV) (*n* = 42), Human alphaherpesvirus 1 (*n* = 33), and Human betaherpesvirus 5 (CMV) (*n* = 30) were the most common viral pathogens.

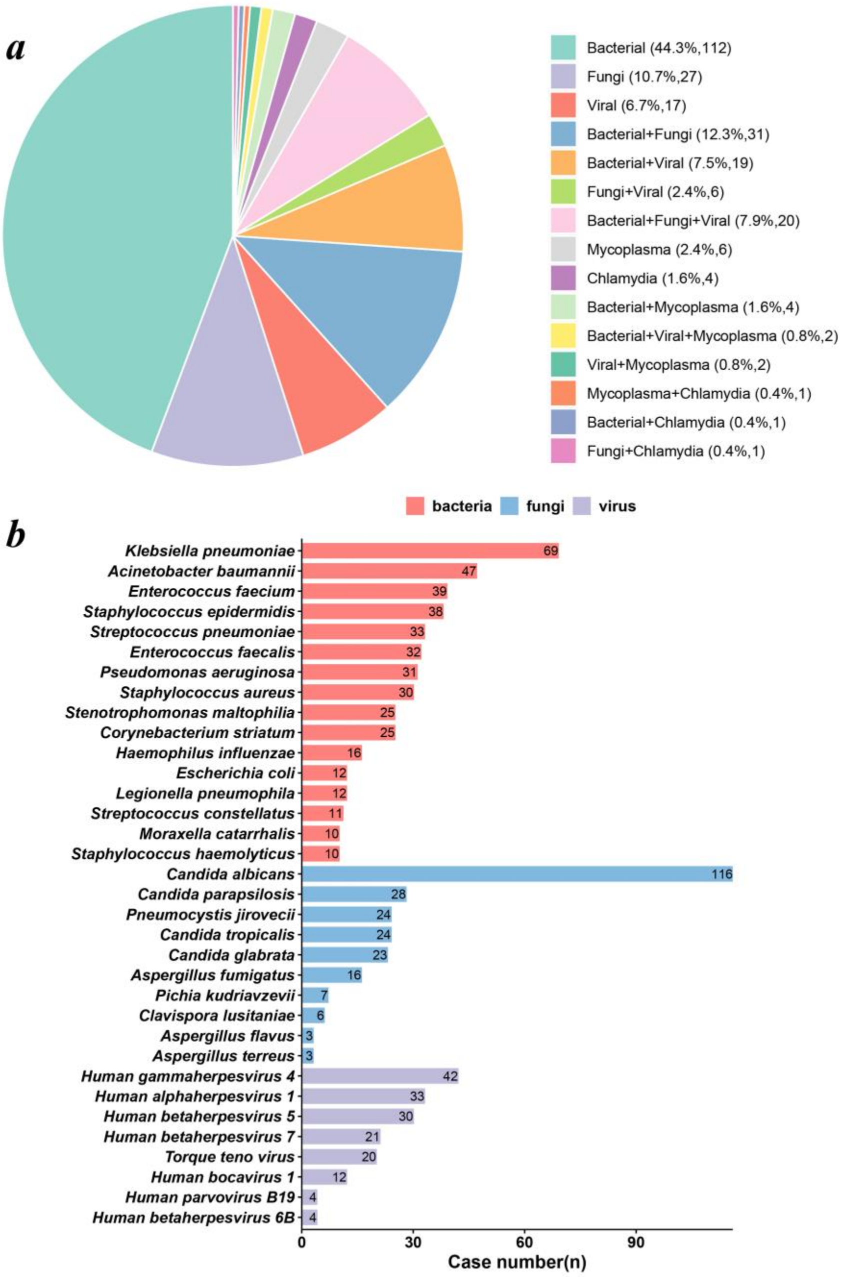

Figure S1 Characteristics of pulmonary infection and mNGS detection results. a, Different kinds of infections. b, mNGS detection.
